# Supplementary material for: Long-term efficacy of mandibular advancement devices in the treatment of adult obstructive sleep apnea: A systematic review and meta-analysis
Source: PLoS One. 2023 Nov 28;18(11):e0292832. doi: 10.1371/journal.pone.0292832 (PMC10684110; doi:10.1371/journal.pone.0292832)
Supplement: Attachment — Submitted filename: respond to reviewers.docx [file pone.0292832.s002.docx]

Dear Editor and Reviewers,

Thank you for your comments concerning our manuscript entitled “Long-term efficacy of mandibular advancement devices in the treatment of adult obstructive sleep apnea: a systematic review and meta-analysis” (PONE-D-23-24787). We greatly appreciate all the comments provided on our paper. We have thoroughly reviewed each comment and made revisions accordingly with the hope of meeting approval. The revised sections have been clearly marked in the article. The main correction and response to the reviewers' comments are outlined below:

Responds to the reviewers’ comments:

**Reviewer 1:**

**1. The paper is a classical technical Meta Analyses of breathing sleep related findings on efficacy of MAD; oral appliance used to manage sleep apnea.**

**It is of interests although it is confirmatory in nature. The presentation of most relevant outcome, hypoxia, should be added to the Abstract. It is a little odd, the most relevant outcomes for health and survival plus debate on compliance (CPAP over MAD) are eluded…. In the actual format, the paper is outdated; please be in line with actual debate and literature.**

**Respond:** Thank you so much for sharing your valuable suggestions with us. We are truly grateful for your input and have taken your feedback to heart. We have made some important changes to the article based on your suggestions, including incorporating synthesized outcomes related to RDI, sleep efficiency, arousal index, blood pressure, and functional abilities as represented by FOSQ in the meta-analysis. We have also included comparisons of dropout rates between MAD and CPAP based on four RCTs to address the issue of compliance. These changes will go a long way in improving the readability and overall quality of the article. Thank you again for your help in this matter. Please find the revised content on Page 12.

**2. Missing information (probably not available in papers revised) on comorbidities, medications used are missing. If not available add to Discussion for suggestions of future studies.**

**Respond:** Thank you for providing your valuable feedback. Most articles did not adequately address outcomes related to comorbidities and medication use, as you predicted. Two articles reported the change in blood pressure after long-term treatment with MAD, and we have added the outcome to the manuscript (Page 13, Line 3). As per your comment, we have included recommendations for further research in the discussion section. Please find the revised content on Page 16.

**3. Similarly, most studies using MAD did not assess compliance objectively, new technological development allow such estimation. It should be also add to Discussion.**

**Plus, some controversy on risk of side effect, add to Discussion. This was in Introduction but absent of Discussion.**

**Respond:** We truly appreciate your suggestions. The addition of objective compliance and side effects of MAD use to the discussion has certainly made it more informative and valuable. Thank you for your contribution. Please find the revised content on Page 15.

Thank you for taking the time to share your thoughts with us. Your input is valuable and we sincerely appreciate it.

**Reviewer 2:**

**1. 1. Table 1. The authors describe a mixture of values regarding the number of patients in the original samples and the analysed number of patients, which makes column 4 (N) and column 10 (Dropouts) difficult to understand and compare. All these data have to be controlled and reported in a logic manner. The numbers reported have to be better explained in the Table, since it becomes impossible to understand that, for example the Baldini et al. retrospective study (reference 46) describes N=117 and Dropouts as 327 patients. It was actually, originally 444 patients, 327 dropped out or denied participation and the remaining 117 patients were evaluated. In the RCT studies, for instance Tegelberg et al. (reference 28) these figures means that 74 patients were originally included, 19 dropped out, leaving 55 for analysis. The Table must be designed so that the data become understandable. The authors also need to include, not only patients who actually dropped out, but also those who were excluded of other reasons, for instance the Rose et al. study (reference 38) included 86 patients, only 26 were evaluated, but the authors only describe 23 patients as drop-outs. These 23 patients constituted only a subgroup of a subgroup. The data should be described in 4 columns according to:**

**Original sample - Dropouts (N +%) - Analysed (N, %) - Women/Men (%) in analysed sample.**

**Respond:** Thank you so much for your suggestions. We have adjusted the table based on your advice to improve readability. Please find the revised content on Pages 7 to 10.

**2. The authors should comment something about the exclusion criteria in these included studies in the discussion, since the studies do not include patients with for instance; an urgent need for OSA-treatment because of comorbidities or dental problems. This influences comparisons with results from long term outcomes for PAP, in addition a much more effective treatment in terms of AHI reductions.**

**Respond:** We appreciate your suggestion and understand the importance of considering exclusion criteria when estimating MAD efficacy. Thank you for bringing this to our attention. Please find the revised content on Page 15.

**3. The authors should include more about the influence of the high drop-out rates for the interpretation of the present findings as well as include something about that in the conclusion. It is not enough, to just mention this in the limitations.**

**Respond:** We appreciate your suggestions. It was a productive discussion where we highlighted the impact of high dropout rates on the overestimation of MAD treatment. It's important to consider all factors that can affect the efficacy of the treatment, as you commented. Please find the revised content on Page 15.

**4. On page 12, lines 17-19, the authors write: “However, previous studies found that there was no difference between MAD and PAP in improving subjective and objective sleepiness, quality of life, cognitive function, and blood pressure.” This statement probably only applies to mild to moderate cases regarding blood pressure (Randerath, W., Verbraecken, J., et al. 2021) and there also some results that PAP is more effective on daytime sleepiness in more severe cases (Sharples, L.D., Clutterbuck-James, A.L., et al. 2016). Please modify this statement, also with respect to point 3 above, where it must be mentioned that many patients are excluded in the MAD studies.**

**Respond:** We appreciate your valuable input. We have taken note of your suggestion and updated our information to provide a clearer understanding of how MAD can effectively address comorbidities depending on the severity of OSA, as well as its long-term benefits. Thank you for helping us improve our content. Please find the revised content on Page 16.

**5. There are more longer term studies available that should be included, if they meet the quality criteria for the present review (Doff, M.H., Hoekema, A., et al. 2013, eSilva, L.O., Guimaraes, T.M., et al. 2021, Gauthier, L., Laberge, L., et al. 2011, Haviv, Y., Bachar, G., et al. 2015)**

**Respond:** Thank you for the comment. The studies conducted by Doff et al and Gauthier et al were added to the included studies. We sincerely apologize for our mistake.

The study conducted by Haviv et al in 2015 (doi: 10.1111/odi.12291) was excluded because the follow-up period was “24 months (range, 5–76 months)”, indicating some participants were followed less than a year. The study conducted by e Silva et al in 2021 (doi: 10.1007/s11325-020-02183-1) and that conducted by Luz et al in 2023 (doi: 10.1007/s11325-022-02694-z) had the same study design and participants, and these two studies shared the same results of before and after-MAD treatment PSG parameters. Therefore, we only included the latest manuscript of Luz’s study.

Other studies are high-quality review articles, which were excluded from the meta-analysis but were added to the discussion where appropriate. Your suggestions have been greatly appreciated.

We’re grateful for your insightful input. Thank you so much for sharing your thoughts with us!
